# Practical Online Monitoring of Ionic Liquid Fiber Welding Solvent

**DOI:** 10.1021/acsomega.1c03122

**Published:** 2021-08-18

**Authors:** Andrew Horvath, Jaclyn Curry, Luke M. Haverhals, Scott K. Shaw

**Affiliations:** † University of Iowa, Iowa City, Iowa 52242, United States; ‡ Natural Fiber Welding, Incorporated, Peoria, Illinois 61625, United States; § Bradley University, Peoria, Illinois 61625, United States

## Abstract

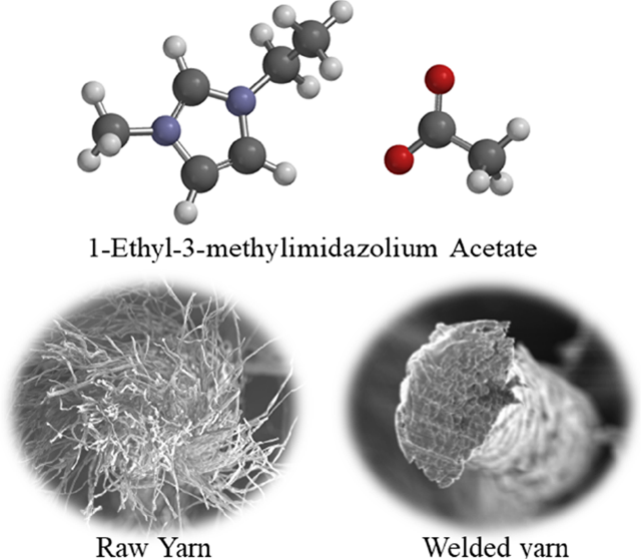

Ionic liquids (ILs) are becoming important solvents in commerce, but monitoring their purity and performance in industrial applications presents new challenges. Fiber welding technology utilizes ILs to mold and shape natural fibers (cotton, hemp, flax, silk, and wool) into morphologies that are typically attained only using synthetic, petroleum-based non-biodegradable plastics. The result is an atom-efficient process that up-converts fibrous substrates to value-added products and materials. A key aspect of bringing this and other IL-enabled technologies to market relies on efficient monitoring and recycling of IL-based solvents. Implementing online IL quality monitoring enhances the unit economics of these processes. Here, we characterize and report conductivity measurements, refractometry, and ATR–FTIR spectroscopy techniques for online IL monitoring during an industrial fiber welding process. The online analysis enables more efficient recycling of the IL solvent, increasing the process efficiency and product quality.

## Introduction

Modern textiles are a multitrillion-dollar industry dominated by synthetic materials derived from nonrenewable petrochemicals. Textile production releases harmful byproducts into the environment. These are often not biodegradable, an issue that has come under increasing environmental scrutiny, especially amid recent concerns of microplastic pollutants.^1^ Natural yarns, such as cotton or wool, have been replaced over time with low-cost and customizable synthetic yarns made of synthetic polymers such as polyacrylonitrile, polyesters, and polyamides under trade names Orlon, Dacron, and Nylon, respectively. Sustainable production of textiles requires development of high-performance and low-cost cellulosic yarns.

**Figure 1:**
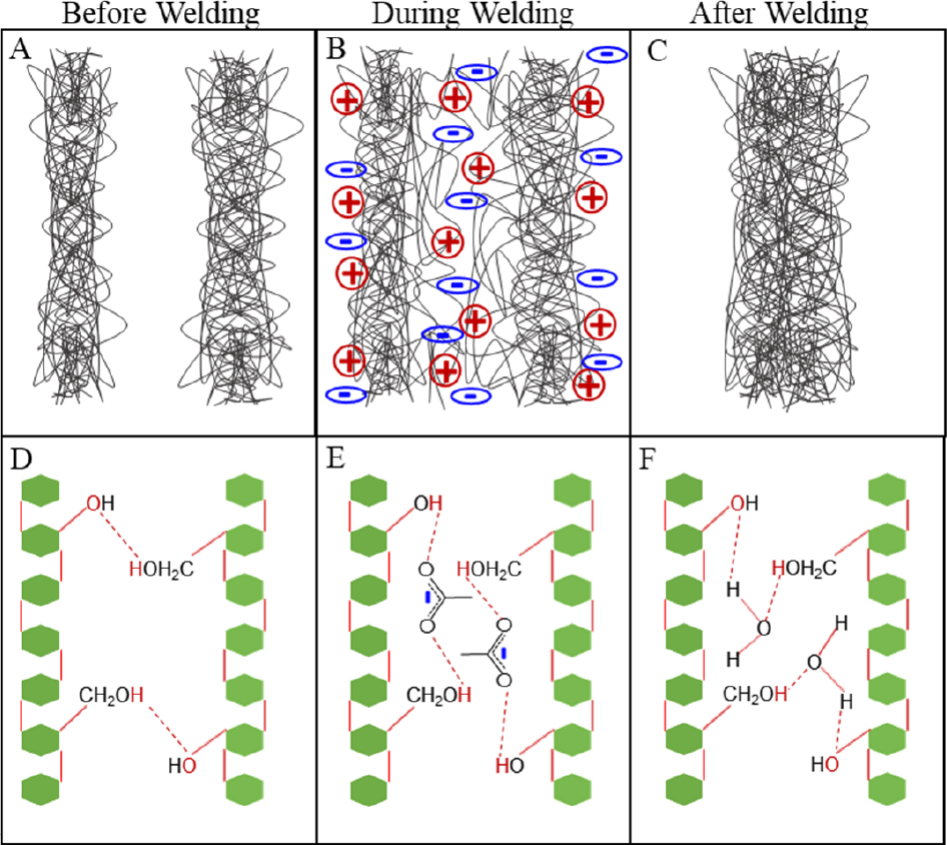
A schematic depicting the fiber welding process. Panels (A)–(C) show bundles of cellulose fibers before (A), during (B), and after (C) the fiber welding process. A limited amount of welding medium is added to a fibrous material. The IL disrupts the hydrogen bonding between polymers. Polymers at fiber surfaces swell and interact with the material from adjacent fibers. After welding, new hydrogen bonds are established between adjacent fibers, which contract into a new fibrous material. Panels (D)–(F) show hydrogen bonding between two individual cellulose polymers before welding (D), during welding (E), and after quenching the reaction with water (F).

The performance metrics of cellulosic yarns are driven by the morphology and length of their fibers or staples.^2−4^ Potential feedstocks for cellulosic yarns include cotton, hemp, flax, silk, and wool. These sustainable sources include many short fibers, which are economically undesirable and problematic in manufacturing. In order to be useful, the short fibers should be converted into longer, tighter strands. This is challenging because natural cellulose fibers are highly ordered and bonded together by a strong hydrogen bonding network (Figure 1), which makes them insoluble in traditional aqueous or organic solvents.^5^ However, the Rogers group showed that certain ionic liquids (ILs) are able to dissolve cellulose by disrupting the hydrogen bonding network.^6^ In particular, the IL 1-ethyl-3-methylimidzolium acetate ([EMIM][OAc]) has been shown to be an optimal solvent for the controlled dissolution of cellulose within cotton yarns.^7−9^

**Figure 2:**
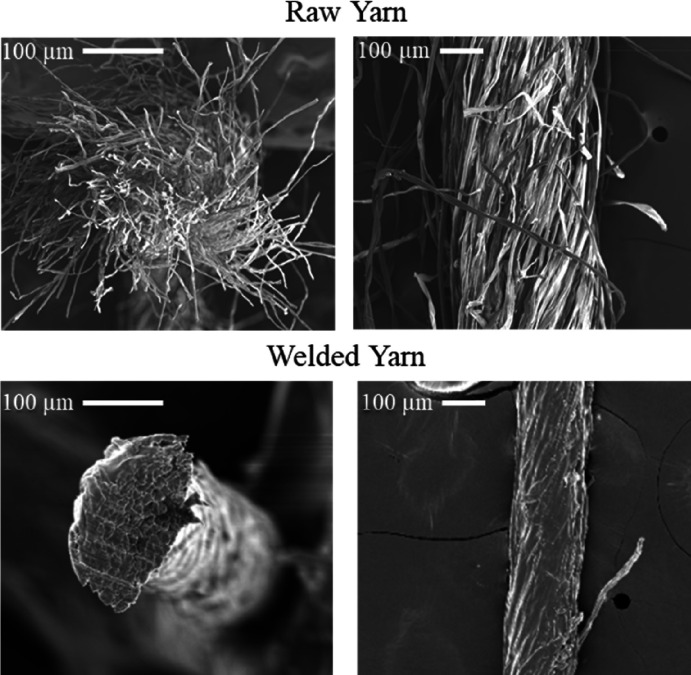
Cross-sectional (left) and top-down (right) SEM images of raw yarn (top) and welded yarn (bottom).

Figure 1 shows a schematic for how the hydrogen bonding networks within cellulose interact with the acetate anion, ultimately allowing “fiber welding”, which creates a high-performance customizable yarn, even from suboptimal short-fiber starting materials, without the use of petrochemicals. The process of fiber welding does not involve full dissolution. Instead, cellulose at the exterior of fibers is swollen by IL [e.g., 1-ethyl-3-methylimidazolium acetate ([EMIM][OAc])] and disrupts the hydrogen bonding network, allowing the cellulose polymer strands to align and fuse to the neighboring fibers.^5,10^ After appropriate time, the cellulose fibers undergo a reconstitution step wherein the welding reaction is quenched and the cellulose returns to a crystalline form. Figure 2 shows a cross section of yarn before (top) and after (bottom) the welding process. Changes in the physical structure of the yarns are clearly visible. The fiber welding process proceeds first with the deliberate introduction of a controlled amount of welding medium into the yarn. Bundles of fibers within the yarn are swelled by the welding medium, while individual fiber strands on the surface of each bundle are partially dissolved, forming a kind of cellulose gel.^5,7,11^ While in the partially dissolved state, the fiber bundles are much more mobile, which brings them into close proximity to each other. Quenching this process at the appropriate time results in new arrangements of hydrogen bonds between fiber strands and an overall contraction in the yarn diameter.^5,7,11,12^

The fiber welding process allows for the production of yarns with tunable physical properties and results in an improvement in the mechanical properties of the yarn. These include an increase in tensile strength, a decrease in yarn diameter, and a decrease in hairiness of the yarn, which leads to more breathability in finished fabrics. Figure S1 shows differences in the mechanical properties of the yarn before and after the fiber welding process. The average breaking strength of an example yarn increases from 415 ± 29 cN with an elongation of 6.3 ± 0.5% before welding to 568 ± 46 cN with an elongation of 4.6 ± 0.4% after welding. Furthermore, the yarn’s diameter decreases from 0.34 ± 0.02% to 0.28 ± 0.03% as a result of the welding process. The full benefits of natural fiber welding have also been previously characterized by Trulove and co-workers and are outside the scope of this study.^5,13,14^

Recent research into fiber welding has identified several additional candidate ILs for use in fiber welding including alkylimidazolium-, alkylpyrolidinium-, and alkylammonium-based liquids with a variety of anion combinations.^6^ It was once thought that ideal ILs for biomass processing required a hydrogen bond donating a cation and a chaotropic anion (capable of disrupting hydrogen bonding). More recent work has established that the dominant factor in the efficiency of an IL for biomass dissolution is simply the presence of the chaotropic anion, while the choice of cation has a limited effect. Trulove and co-workers used confocal fluorescence microscopy to show that non-hydrogen-bond donating ILs such as alkylpyrolidinium acetate ILs exhibited similar efficiencies when used in the fiber welding process.^13^ It is desirable to use [EMIM][OAc] in the fiber welding process for several reasons. It has a relatively high ability to dissolve cellulose (20 wt %), a low viscosity and melting point relative to other ILs, low toxicity, and low cost of production.^15^

In order for [EMIM][OAc] to be effective at fiber welding, the water content should be relatively low, typically <1% by mass.^16^ This is due to water’s preference to solvate acetate ions, which precludes the disruption of the cellulose hydrogen bonding network. The water used to wash the IL from welded fibers can be removed from the welding medium by distillation or industrial film evaporators.^17^ Typically, short path distillation followed by centrifugation and filtration is employed to minimize the recycling time and remove water-soluble contaminants that precipitate upon dehydration of the welding medium. The temperatures employed, typically in excess of 120 °C, can result in thermal degradation of the IL, decreasing efficiency in subsequent welding cycles.^17,18^ Flexibility in the welding process can be achieved by adding organic cosolvents to the IL. The high viscosity of pure [EMIM][OAc] (139 cP) limits its diffusion into the yarn, slowing the welding rate. The addition of a cosolvent to increase the diffusion of IL into the yarn can be done.^19^ Dimethyl sulfoxide (DMSO) is a polar aprotic solvent that acts as a cosolvent reducing the welding medium viscosity and swelling the cellulose fibers, leading to a higher quality and faster processing.^20−22^

Welding reproducibility across material types and qualities is essential for textile applications. The extent of welding is a measure of macroscopic and microscopic properties of the reconstituted cellulose within yarn. These include the strength, elongation, and hairiness of the yarn as well as a visual inspection of a cross section of the yarn via SEM, as shown in Figure 2. These parameters are tuned by controlling the welding solution composition, application rate, and the yarn’s residence time in the solution. Accurate, continuous monitoring of the welding medium composition is required during processing. Monitoring the concentrations of IL, DMSO, and water in recycled solutions is critical for maintaining quality control standards.

The extent to which a yarn is welded can be controlled by adjusting the contact time between the IL and the yarn. The welding reaction is quenched by rinsing the yarn with water. Water halts the welding reaction by displacing the imidazolium and acetate ions from the yarn and by forming hydrogen bonds to cellulose-free acetate anions in solution. Maximizing the recovery and reuse of ILs is important for the economic viability of fiber welding processes.

The dehydration of [EMIM][OAc] is particularly difficult due to water’s ability to form strong hydrogen bonds with the acetate anion. The negligible vapor pressure of ILs makes them ideal candidates for dehydration by distillation; however, this can lead to the thermal degradation of the IL without appropriate control of recycling processes. Monitoring rinsing solutions is helpful to aid the efficiency of recycling processes.

This report presents an efficient method to continuously monitor IL concentration in the rinsing solution created during “welding” processes. We describe conductivity measurements, refractometry, and vibrational spectroscopy measurements that provide critical information to maximize recovery of IL from aqueous/organic mixtures.

## Results and Discussion

**Figure 3:**
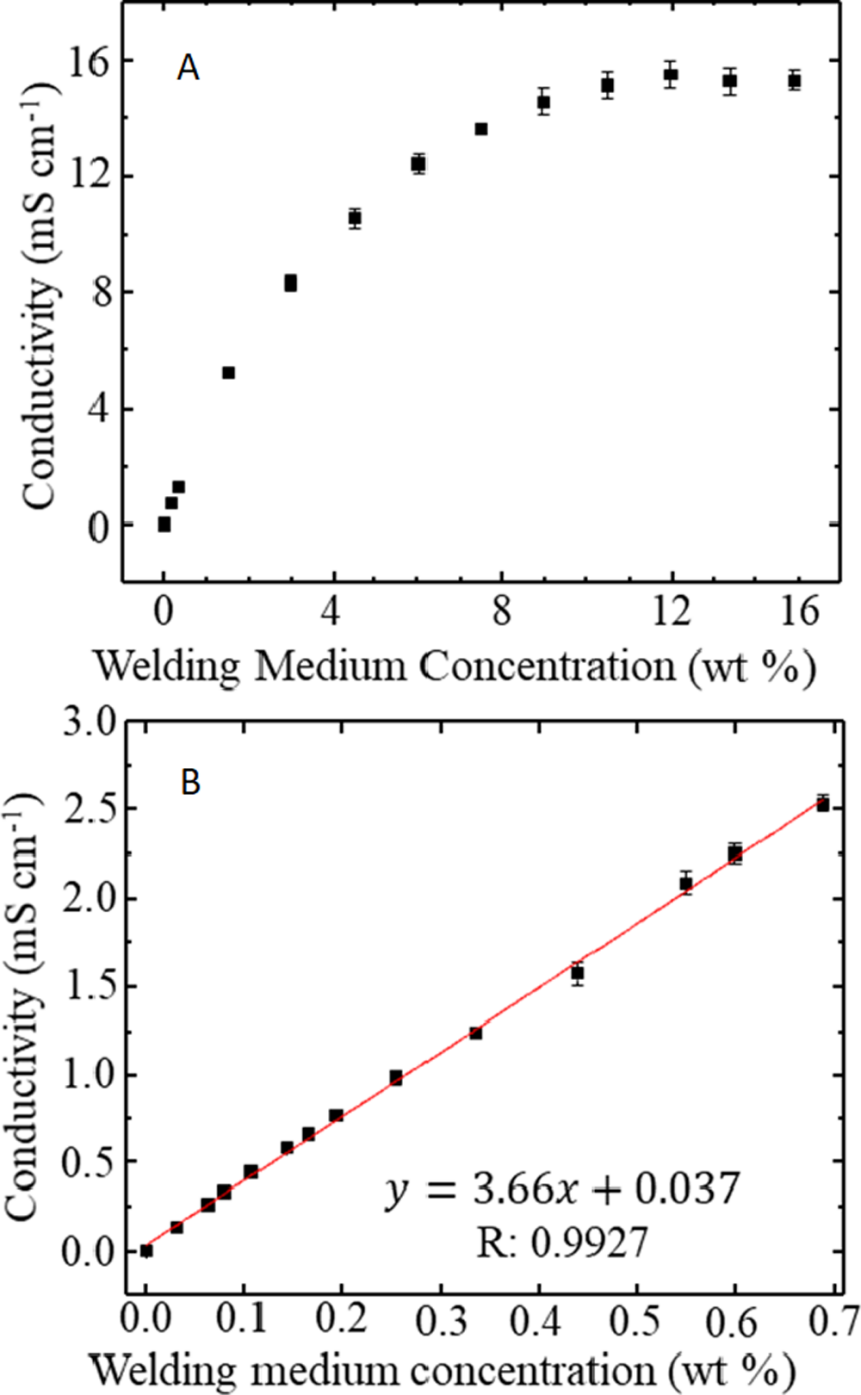
Plots of the dynamic range (A) and linear range (B) of conductivity for varying wt % of the welding medium (IL/DMSO) in water. Data points (black squares) represent replicate measurements where *n* ≥ 3. Standard deviation error bars are contained within the size of the data points. The red line is a linear fit to the individual data points.

Consistent and accurate monitoring of the aqueous/organic/IL mixture rinse solution is critical for ensuring the efficient recovery and recycling of IL. Currently, the [EMIM][OAc] concentration is quantified by a combination of conductivity and refractometry. Figure 3 shows the dynamic (A) and linear (B) ranges for the conductivity of a solution of water mixed with varying concentrations of welding medium. The dynamic range of this measurement extends to approximately 10 wt % [EMIM][OAc], and the linear range extends to approximately 0.7 wt %. This is a very limited linear range and does not reach the 20–40 wt % at which ILs are used in production. Conductivity values are also affected by contaminants that are washed from the yarn during welding and concentrated in the rinsing solution. These include minerals, waxes, and organic acids present in many raw or recycled yarns. An inaccurate conductivity measurement can lead to inaccurate IL concentrations, resulting in changes in the properties of the finished product as well as inefficiencies in IL recovery and recycling. Therefore, conductivity is not desirable for online analysis in manufacturing.

**Figure 4:**
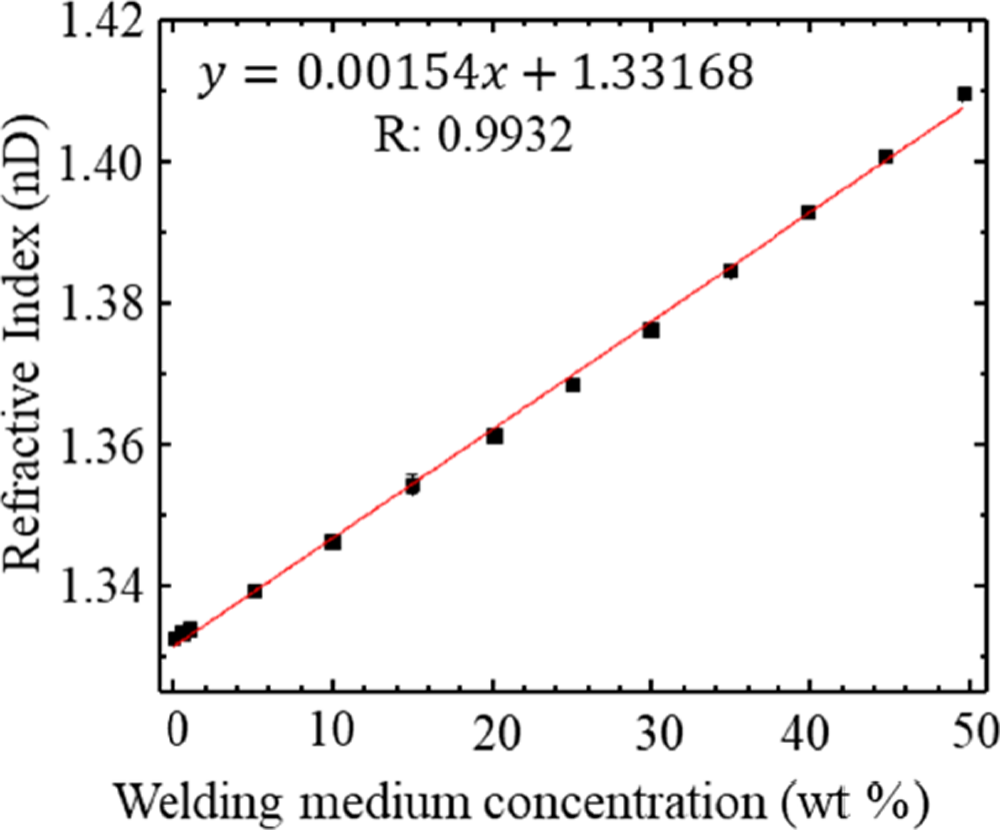
Plot of the refractive index for varying wt % of the welding medium (IL/DMSO) in water. Data points (black squares) represent replicate measurements where *n* ≥ 3. Standard deviation error bars are contained within the size of the data points. The red line is a linear fit to the individual data points.

Refractive index varies with solution composition and has potential for use as a contactless metric for determining IL concentration during the fiber welding process. Figure 4 shows the refractive index of samples containing varying amounts of the welding medium in water. The refractive indices of neat DMSO and [EMIM][OAc] at 20 °C were measured to be 1.479 and 1.502, respectively, which agree well with the established values^23,24^ but provide a slim margin for differentiating the two. Quantifying differences between water and a mixture of DMSO and IL is simpler because the refractive index of water is 1.333, sufficiently different than the refractive indices of the other species. Additionally, the calibration curve of the refractive index and welding medium concentration remains linear up to a concentration of 50% w/w of water in the welding medium (IL/DMSO). Refractive index measurements unfortunately share similar susceptibility of conductivity measurements to common interferences, e.g., those from dissolved species in solution that lead to errantly high refractive index readings. This in turn leads to inaccurate quantification of IL concentration and complicates IL recovery. Figure S2 describes in detail how the presence of interferences leads to incorrectly high concentrations calculated by the refractive index.

**Figure 5:**
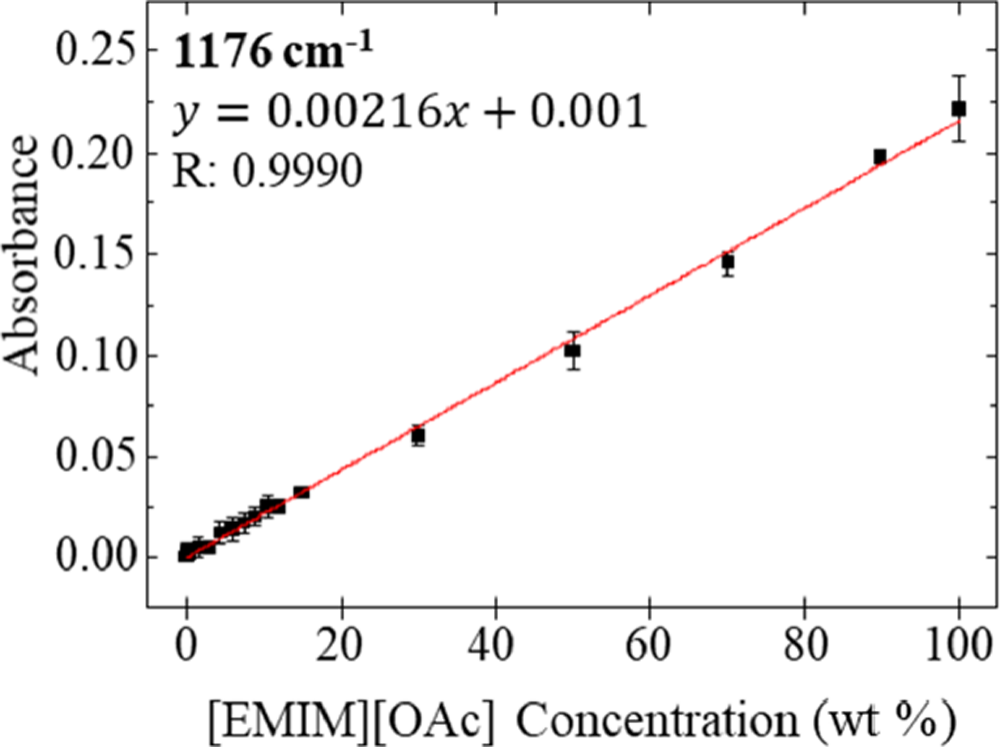
Plot of absorbance of the peak at 1176 cm^–1^ for dilutions of [EMIM][OAc] in DMSO. Water content of each sample was determined to be <1%. Data points (black squares) represent replicate measurements where *n* ≥ 3. Standard deviation error bars are contained within the size of the data points.

Conductivity and refractive index measurements aid in the determination of welding medium composition; the primary drawback to both methods of analysis is that contamination by species present in raw yarn can lead to inaccurate results. However, infrared spectroscopy provides simultaneous measurements of solution composition and concentration. Figure S3 shows a series of spectra obtained by attenuated total internal reflectance–Fourier transform infrared (ATR–FTIR) spectroscopy on samples containing varying concentrations of [EMIM][OAc] in DMSO. The spectra show discernible differences in peak height (absorbance), which correlated with the IL concentration in the sample. The vibrational modes present in the high-frequency region from 2700 to 3200 cm^–1^ are indicative of aliphatic C–H stretching modes in the imidazolium cation and DMSO and are difficult to resolve.^25,26^ The peak at 1176 cm^–1^ within the fingerprint region of the spectra corresponding to the C=O stretching mode in the acetate anion is in a relatively clear area of the IR absorption spectrum and varies linearly with [EMIM][OAc] concentration.^27^Figure 5 shows a linear response for this C=O mode intensity vs concentration, suggesting that it is possible to use infrared measurements to monitor IL concentration in the complex welding medium. The water content of each sample was found to be <1% by mass via Karl Fischer titration.

**Figure 6:**
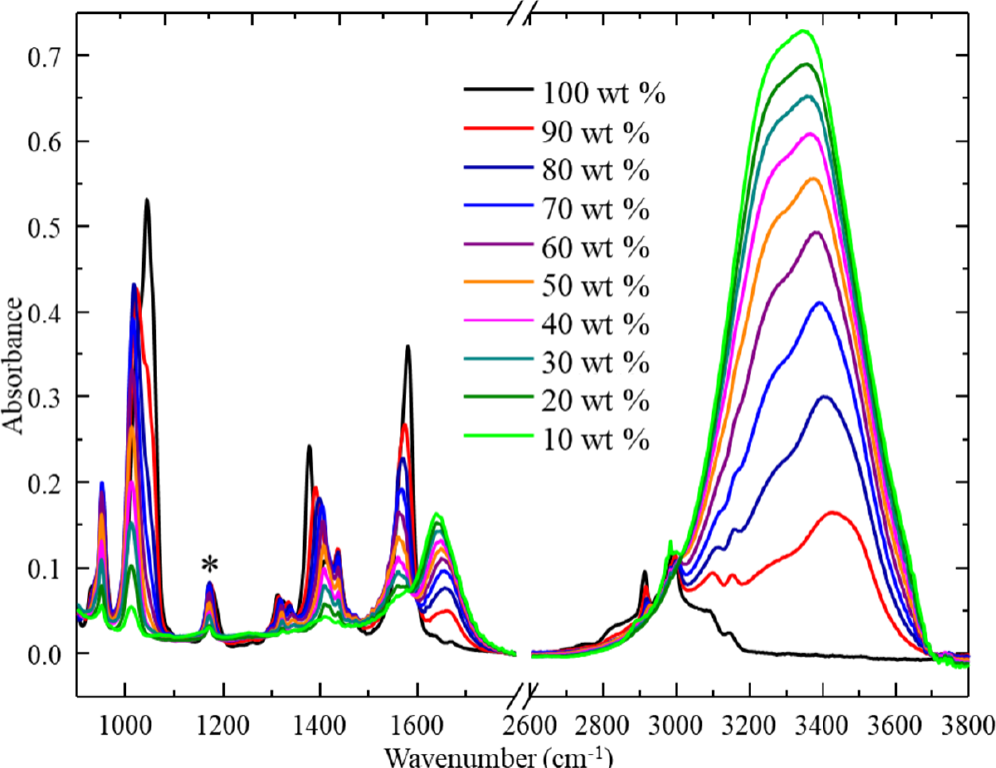
ATR–FTIR spectra of the welding medium in water at various wt % dilutions. The peak at 1170 cm^–1^ was used for concentration analysis. Spectra shown are representative of *n* > 3 prepared samples.

**Figure 7:**
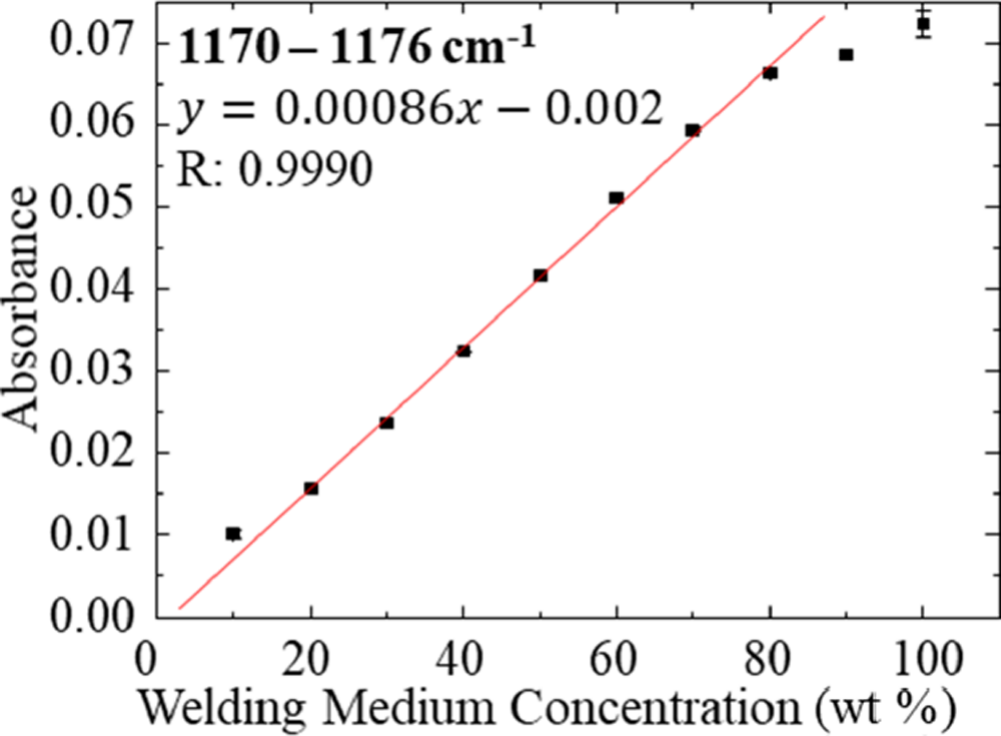
Plot of maximum absorbance of the peak between 1170 and 1176 cm^–1^ for dilutions of the welding medium in water. Data points (black squares) represent replicate measurements where *n* ≥ 3. Standard deviation error bars are contained within the size of the data points.

Figure 6 shows a series of spectra obtained from welding medium at varying concentrations in water. We note that several of the absorption peaks shift as the water content of the solutions increases. This has previously been attributed to solvation affecting the local chemical environment of the IL ion pairs.^28^ In particular, we note that the C=O peak at 1177 cm^–1^ exhibits an initial frequency shift from 1177 to 1170 cm^–1^ upon dilution with water but remains stable at water concentrations greater than 20%, suggesting that bulk solvation conditions may stabilize beyond this concentration. Figure 7 shows that the increasing absorbance of the C=O stretching mode varies linearly with the welding medium concentration in the simulated wash samples. The linear range of this measurement is from 10 to 80 wt % of the welding medium in water. It is noteworthy that the peaks associated with water at 1500–1600 and 3100–3600 cm^–1^ increase as a function of water concentration. These peaks were not selected for welding medium concentration determination because their absorbance does not show a linear dependence on concentration, and they are susceptible to interference by contaminants, including OH groups present in the dissolved cellulose in the welding medium. Figure S4 lists the peak assignments for vibrational modes present in the spectra shown in Figure 6.^29^

**Figure 8:**
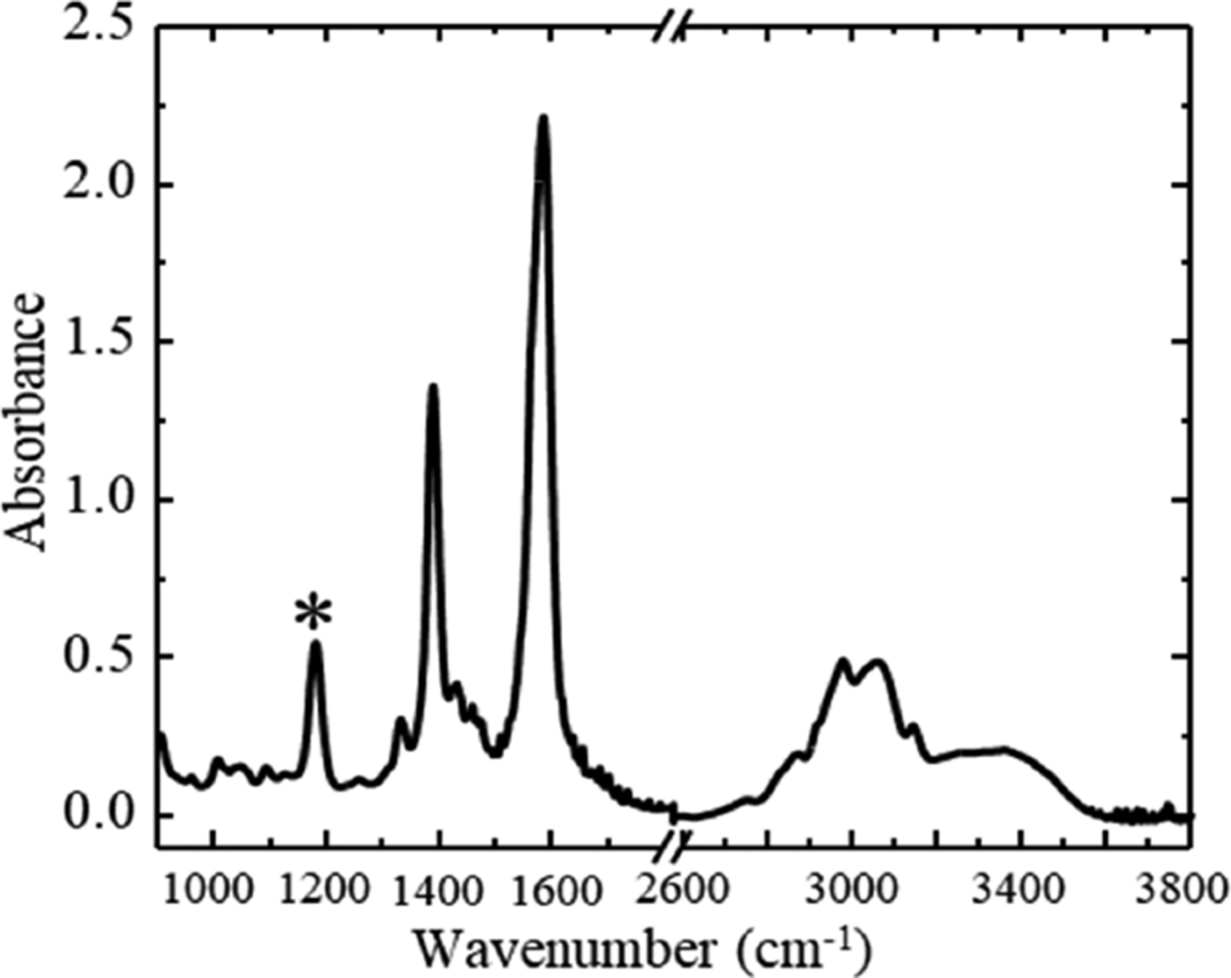
A transmission FTIR spectrum of the recycled welding medium. The peak at 1170 cm^–1^ was used for concentration analysis. The spectrum shown is representative of *n* > 3 prepared samples.

Figure 8 shows a transmission FTIR spectrum collected on [EMIM][OAc] that has been used to process cotton and has been subsequently recovered and reconcentrated. The broad peak at ca. 3400 cm^–1^ corresponds to the O–H stretching mode coming from residual water left in the sample. Water concentration was determined to be 1.7% by Karl Fischer titration. Nonvolatile impurities from the welding process have not been removed, and the IL has not been diluted with DMSO. Therefore, any contaminant species will be most concentrated at this stage. The C=O stretching mode at 1170 cm^–1^ used for quantitation remains in a clear spectral window, confirming that it is a robust peak for IL quantitation even in the presence of process impurities. This implies that online ATR–FTIR spectroscopy using a flow cell configuration is a viable method for continuous, online monitoring of welding medium concentration.

## Conclusions

IL concentration in the rinse solution can be monitored using a combination of conductivity, refractometry, and vibrational spectroscopy. Conductivity measurements measure a small linear range of [EMIM][OAc] concentrations. Measurements of the refractive index of the rinse solution can measure concentrations in solutions of up to 50% w/w welding medium in water but are unable to identify sample components while measuring the concentration. The vibrational modes corresponding to the C=O stretching in the acetate anion are linearly correlated with [EMIM][OAc] concentration and not obscured by other solution components. Therefore, ATR–FTIR spectroscopy is a convenient online method for determining the exact [EMIM][OAc] concentration.

## Experimental Section

### Materials

The IL 1-ethyl-3methylimidazolium acetate ([EMIM][OAc]) (95%, Proionic, Grambach, Austria) and DMSO (Industrial Grade, 99.7%, Gaylord Chemical, Slidell, LA, USA) are used as received. Reverse osmosis (RO) water (30 mS cm^–1^) is produced by an in-house RO system (ESP Water Products, Sunnyvale, TX, USA). Cellulose yarns (Parkdale Mills, Gastonia, NC, USA) are used as received.

### Methods

#### Cellulose Fiber Welding

Natural Fiber Welding, Inc., modifies the properties of cellulose yarns though patented processes. In bench-scale experiments, ca. 20 cm of yarn is soaked in a proprietary welding medium (solution of [EMIM][OAc] and DMSO), the exact composition of which we are unable to share but is encompassed in the testing solutions used here and will not significantly affect the monitoring methods reported in our work. The welding process is quenched by soaking the yarn in a vial of rinse water for a predetermined amount of time, e.g., 5–45 s. After rinsing, the yarn is dried at room temperature on paper towels for ca. 24 h. Production-scale “welding” is performed by automated process equipment and is beyond the scope of this study. During processing, yarns are immersed in a bath of rinse water for a varying amount of time, e.g., 5 to 30 s. Yarn is dried and wound onto cones.

#### Scanning Electron Microscopy

Scanning electron microscopy (SEM) images are acquired using a JEOL instruments JSM 6010PLUS/LA InTouchScope using a tungsten lamp electron source. Cross-sectional and horizontal views of the yarn are collected to compare the degree of welding of the yarn. Yarn samples are cut using a razor blade and laid flat with the cut end angled upward to acquire a cross-sectional image. Yarn samples are sputter-coated with gold (argon purge, 15 nm gold). Images are collected using a 10 kV accelerating energy and a 15 mm working distance. Cross-sectional images use ×200 magnification, and horizontal images use ×100 magnification. Images are representative of *n* > 5 welded yarn samples.

#### Conductivity

Conductivity measurements are made using an Apera Instruments model EC400 parallel plate conductivity probe. The probe is calibrated using three standard solutions (12.88 mS cm^–1^, 84 μS cm^–1^, and 1413 μS cm^–1^). Conductivity of the yarn rinsing solution is measured by soaking a premeasured length of dry yarn in distilled water for 24 h, followed by measuring the conductivity of the soaking water. Solutions containing [EMIM][OAc], DMSO, and H_2_O are diluted with distilled water to keep the conductivity within the calibrated range.

#### Refractometry

Refractive index is determined using a Hanna Instruments HI96800 refractometer. Approximately 0.5 mL of the solution of [EMIM][OAc] in DMSO is dispensed on the sample well so that the entirety of the crystal is submerged. Measurement takes 1.5 s and is shielded from any external light. The refractometer can measure the refractive index of a solution in the range from 1.3300 to 1.5080 ± 0.0005. The refractometer is calibrated using distilled water with a known refractive index of 1.3330.

#### Attenuated Total Reflectance (ATR) FTIR Spectroscopy

Infrared spectroscopy measurements are completed using a Thermo-Scientific Nexus 470 Fourier transform infrared spectrometer and a Smart Endurance ATR accessory with a 0.75 mm 42° single reflection diamond-faced ZnSe prism. A deuterated triglycine sulfate (DTGS) detector is used to collect spectra from 400 to 4000 cm^–1^. A background spectrum is taken prior to each sample spectrum. Sample spectra are collected by covering the exposed ATR crystal with ca. 0.5 mL of the solution containing known weight percent of [EMIM][OAc] in DMSO. Each background and sample spectrum are collected using 32 scans at a 4 cm^–1^ resolution. The sample chamber is purged with dry nitrogen gas to reduce water vapor. Vibrational mode analysis is completed using OriginLab software.

## Supporting Information Available

Mechanical properties of cotton yarn before and after the natural fiber welding process (Figure S1), a plot of refractive index vs wt % of [EMIM][OAc] in a solution containing 1 wt % water with the balance being DMSO (Figure S2), ATR–FTIR spectra of [EMIM][OAc] in DMSO in various weight percent dilutions (Figure S3), and peak assignments corresponding to IR spectra of welding solvent–water samples (Figure S4) (PDF)
