# Supplementary material for: Practical Online Monitoring of Ionic Liquid Fiber Welding Solvent
Source: ACS Omega. 2021 Aug 18;6(34):22367–73. doi: 10.1021/acsomega.1c03122 (PMC8412937; doi:10.1021/acsomega.1c03122)
Supplement: Supplementary file 1 — ao1c03122_si_001.pdf [file ao1c03122_si_001.pdf]

Supporting Information for:

Title: Practical Online Monitoring of Ionic Liquid Fiber Welding Solvent

Authors: Andrew Horvath[1]; Jaclyn Curry[1]; Luke Haverhals;[2,3] Scott K. Shaw[1*]

Institutions: University of Iowa[1]; Natural Fiber Welding[2]; Bradley University[3]

Figure S1. Mechanical properties of cotton yarn before and after the natural fiber welding process.
Figure S2. A plot of refractive index vs. %wt of [EMIM][OAc] in a solution containing 1 %wt water with the balance being DMSO
Figure S3. ATR-FTIR spectra of [EMIM][OAc] in DMSO in various weight percent dilutions
Figure S4. Peak assignments corresponding to IR spectra of welding solvent-water samples.

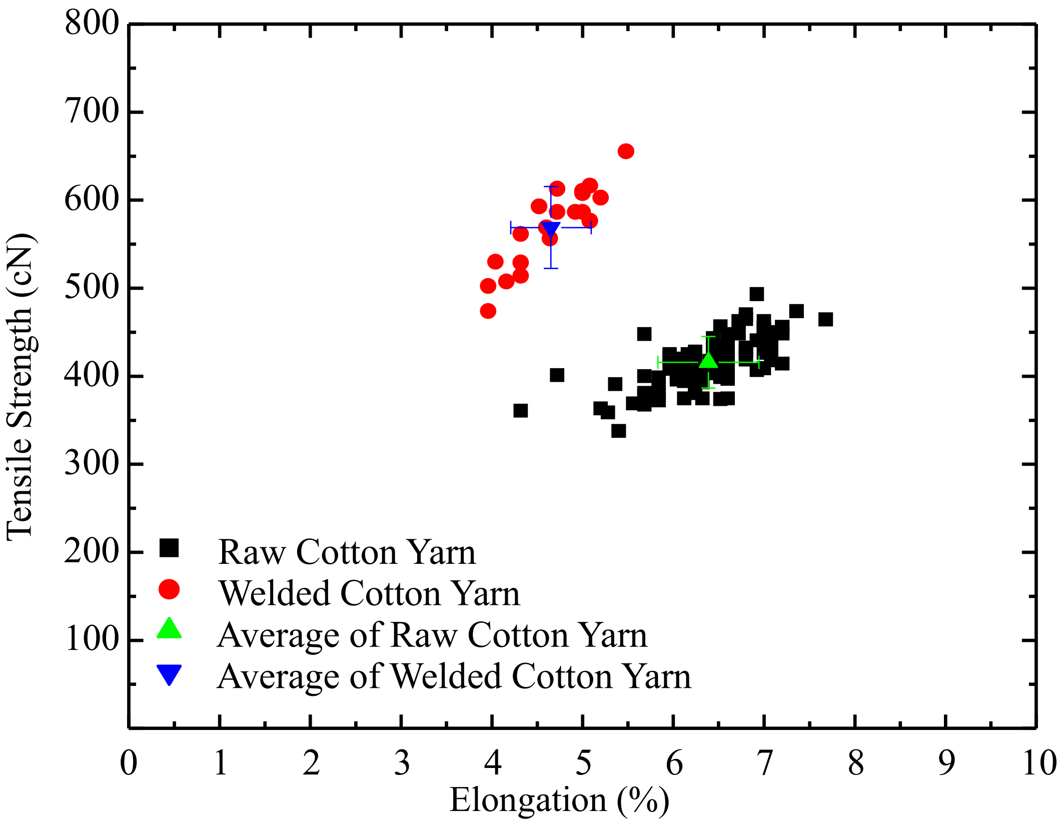

Figure S1. A plot of tensile strength vs elongation at failure of a cotton yarn before and after the natural fiber welding process. Data points (black squares and red circles) are collected from n>20 trials. Green and blue triangles and associated error bars represent the average and standard deviation of the red and black data clouds respectively. Yarn is stretched by an automatic tensile testing machine until it breaks.

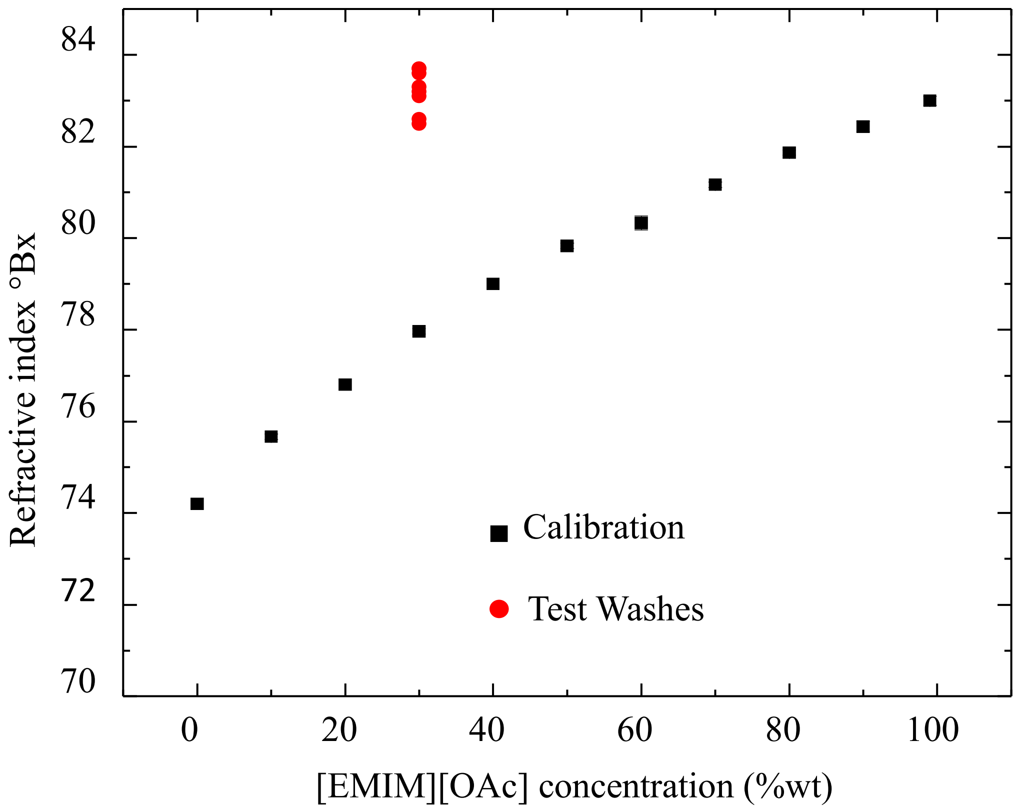

Figure S2. A calibration plot of refractive index in degrees brix vs. %wt of [EMIM][OAc] in a solution containing 1 %wt water with the balance being DMSO. Calibration data points (black squares) represent replicate measurements where n≥3. Standard deviation error bars are contained within the size of the data points. Test wash (red circles) represent samples of wash solution taken from different yarn lots. Each yarn lot was prewashed using varying methods prior to welding.

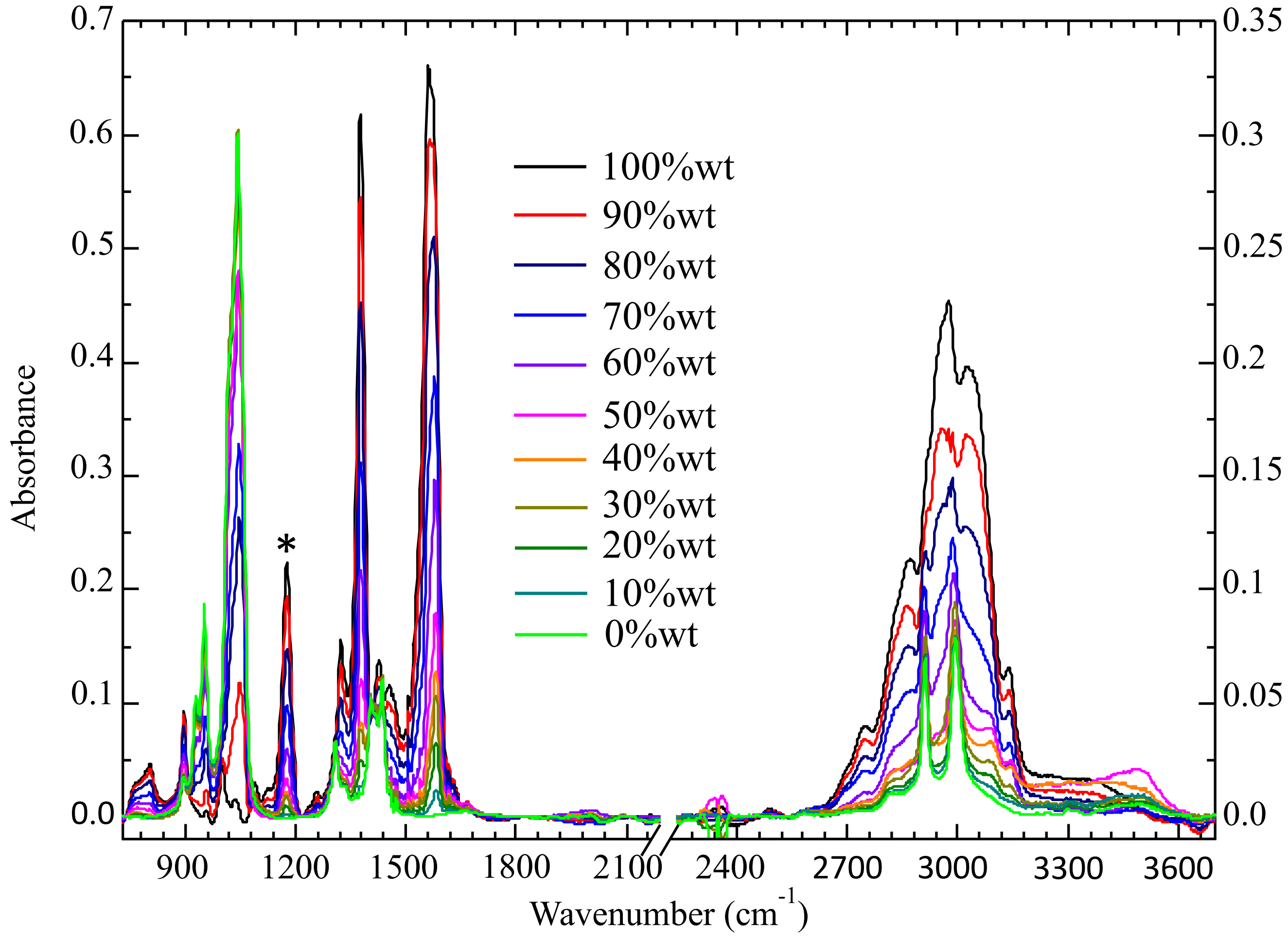

Figure S3. ATR-FTIR spectra of [EMIM][OAc] in DMSO in various weight percent dilutions. Different scales for the region between 800 cm^{-1} and 2100 cm^{-1} and the region between 2400 cm^{-1} and 3600 cm^{-1} are shown on the left and right axes of the plot respectively. Spectra shown are representative of n>3 prepared samples.

**Figure S4.** Peak assignments corresponding to IR spectra of welding solvent-water samples.	
**Vibrational Frequency (cm^{-1})**	**Assigned Vibrational Mode**
3450	O-H Stretching
3156	Symmetric Aromatic C-H stretching
3087	Asymmetric Aromatic C-H stretching
3000	Symmetric CH_2 Stretching
2914	Asymmetric CH_2 Stretching
1662	O-H Bending
1580	Aromatic C=N stretching
1439	CH_2 Bending
1390	Symmetric CH_3 Bending
1331	Symmetric C=O Stretching
1308	Asymmetric CH_3 Bending
1174	Asymmetric C=O Stretching
1027	S=O Stretching
955	CH_3 Rocking
